# Design, Fabrication, and Applications of SERS Substrates for Food Safety Detection: Review

**DOI:** 10.3390/mi14071343

**Published:** 2023-06-30

**Authors:** Ding-Yan Lin, Chung-Yu Yu, Chin-An Ku, Chen-Kuei Chung

**Affiliations:** Department of Mechanical Engineering, National Cheng Kung University, Tainan 701, Taiwan

**Keywords:** food safety, sensing, SERS, nanofabrication, nanostructures

## Abstract

Sustainable and safe food is an important issue worldwide, and it depends on cost-effective analysis tools with good sensitivity and reality. However, traditional standard chemical methods of food safety detection, such as high-performance liquid chromatography (HPLC), gas chromatography (GC), and tandem mass spectrometry (MS), have the disadvantages of high cost and long testing time. Those disadvantages have prevented people from obtaining sufficient risk information to confirm the safety of their products. In addition, food safety testing, such as the bioassay method, often results in false positives or false negatives due to little rigor preprocessing of samples. So far, food safety analysis currently relies on the enzyme-linked immunosorbent assay (ELISA), polymerase chain reaction (PCR), HPLC, GC, UV-visible spectrophotometry, and MS, all of which require significant time to train qualified food safety testing laboratory operators. These factors have hindered the development of rapid food safety monitoring systems, especially in remote areas or areas with a relative lack of testing resources. Surface-enhanced Raman spectroscopy (SERS) has emerged as one of the tools of choice for food safety testing that can overcome these dilemmas over the past decades. SERS offers advantages over chromatographic mass spectrometry analysis due to its portability, non-destructive nature, and lower cost implications. However, as it currently stands, Raman spectroscopy is a supplemental tool in chemical analysis, reinforcing and enhancing the completeness and coverage of the food safety analysis system. SERS combines portability with non-destructive and cheaper detection costs to gain an advantage over chromatographic mass spectrometry analysis. SERS has encountered many challenges in moving toward regulatory applications in food safety, such as quantitative accuracy, poor reproducibility, and instability of large molecule detection. As a result, the reality of SERS, as a screening tool for regulatory announcements worldwide, is still uncommon. In this review article, we have compiled the current designs and fabrications of SERS substrates for food safety detection to unify all the requirements and the opportunities to overcome these challenges. This review is expected to improve the interest in the sensing field of SERS and facilitate the SERS applications in food safety detection in the future.

## 1. Introduction: Overview of Food Safety Detection

**Figure 1:**
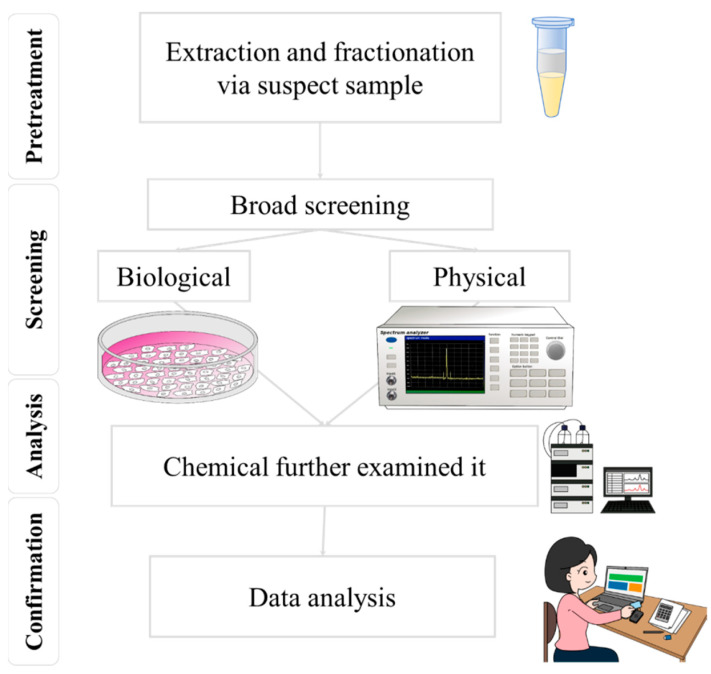
By combining screening and mass spectrometry techniques, the accuracy and efficiency of food safety testing can be effectively improved.

In recent years, there has been an increased awareness of food safety issues, highlighting the importance of food analysis [1]. While chemical methods have been the standard for food safety penalties [2], their high costs, lengthy wait times, and limited coverage have become more apparent. This has led to a renewed focus on developing versatile devices for food safety management, such as rapid screening methods [3]. Rapid screening techniques for food safety primarily utilize biological and physical processes, as shown in Figure 1 [4,5]. In food safety analysis, the bioassay method generally involves analyzing the effects of biological or chemical contaminants on intracellular genes or utilizing cellular metabolic mechanisms, for detection, through an antibody-and-antigen match and a series of biochemical reactions [6]. Instead of focusing on the concentrations of a few select chemicals, bioassays can be a supplementary approach to conventional chemical monitoring methods in the future. They can achieve this by evaluating cumulative effects, such as those caused by mixtures, and addressing the chemicals present at concentrations below the detection limits of chemical analysis [6]. This method can detect harmful substances—rapidly and sensitively—in food, offering advantages such as fast detection, user-friendly operation, and low cost [7]. Nonetheless, the bioassay method presents multiple disadvantages. For instance, if the sample preprocessing has little rigor, it could disrupt the identification of substances, resulting in false positives or negatives, owing to limited specificity [8,9]. Additionally, the precision of the detection outcomes might be influenced by environmental factors within the laboratory, including the integrity of laboratory partitions, temperature, and humidity. Moreover, the bioassay technique demonstrates a particular reliance on the sample’s composition, and its sensitivity might prove less effective than alternative chemical detection methods when examining components.

In contrast, physical methods more directly detect physical properties in food samples, such as optical and acoustic properties, and they have a more comprehensive application range that is not limited by sample type. Physical methods also have a lower dependence on sample components. Laser technology is a physical method that offers benefits such as fast detection speed, high sensitivity, and good selectivity [10]. By controlling the wavelength and power of the laser, it is possible to detect and analyze different components in the sample. Laser technology also allows for non-destructive testing, minimizing damage to the model. Raman spectroscopy, particularly surface-enhanced Raman spectroscopy (SERS) technology, is an emerging physical method that offers ultra-trace analysis capabilities. SERS technology is likely to become the preferred option for preliminary screening under the “screening first and testing later” food safety management approach [11]. The microstructure fabrication and morphology of SERS significantly impact its sensitivity, specificity, selectivity, and anti-interference ability for food safety screening. Raman spectroscopy is a non-destructive spectroscopic technique that enables the rapid and accurate identification and semi-quantification of chemical and biological substances [12]. Mass spectrometry is a technique that allows for the high-sensitivity and high-resolution analysis of complex samples. The combination of Raman spectroscopy and mass spectrometry in food safety detection can enhance the accuracy and efficiency of testing [13]. Leveraging a tandem approach of Raman spectroscopy and mass spectrometry in food safety inspection provides several benefits, including accelerated detection [14], increased precision [13], preservation of sample integrity [12], and real-time surveillance [15]. The initial screening via Raman spectroscopy, coupled with mass spectrometry confirmation and quantification, enhances testing efficiency. These complementary techniques provide diverse chemical insights, corroborating and bolstering the accuracy of results. Importantly, the non-invasive characteristic of Raman spectroscopy and its swift operation allow for timely monitoring to control safety risks. Practical applications span from detecting pesticide residues [16,17] and food additives [18,19], to identifying microbial contamination [20,21] and food poisoning agents [15,22], and to verifying food authenticity. Prompt identification and the quantification of potential hazards assure compliance with safety regulations. Additionally, confirming food genuineness aids in protecting consumer rights [23,24]. In summary, the symbiotic integration of Raman spectroscopy and mass spectrometry substantially improves food safety inspection accuracy and efficiency, thereby playing a pivotal role in fortifying industry standards and safeguarding consumer well-being.

## 2. Current Detection Techniques

Food safety testing techniques have become crucial to ensure the public’s health and safety. We want to detect chemical substances in food, so various techniques must be employed. Consequently, the preprocessing of samples, such as liquid or solid phase extraction, is required, followed by the utilization of three separation methods, namely liquid chromatography (LC), gas chromatography (GC), and capillary electrophoresis (CE) [25]. Subsequently, the separated substances need to be ionized, and different detectors are used for analytical testing. Standard detectors include mass spectrometers (MS) [26], ultraviolet/visible (UV/VIS) spectroscopy, nuclear magnetic resonance (NMR), and near-infrared spectrometry (NIR) [27]. In the food industry, Raman spectroscopy and near-infrared spectroscopy are two common spectroscopic analysis techniques, each with distinct characteristics in terms of setup cost, analysis time, destructiveness to samples, and resistance to interference from moisture. The setup cost for Raman spectroscopy is relatively high, but it has strong resistance to interference from moisture, allowing for an accurate analysis without being affected by water content [28]. In addition, the analysis time for Raman spectroscopy is generally moderate, and it is non-destructive to samples, preserving their integrity [29]. Conversely, the setup cost for near-infrared spectroscopy is low to moderate, the analysis time is fast, and it is non-destructive to samples. However, its resistance to interference from moisture is lower, which may affect the analysis results of food samples with high water content [30]. Therefore, the choice of which technique to use should be determined based on specific analysis needs and conditions [31].

In addition to chemical testing methods, microbiological food safety testing is paramount to ensure the absence of harmful pathogens, spoilage organisms, and other undesirable microorganisms in food products [32]. Microbiological testing methods can be classified into conventional and rapid methods [33]. Coventional methods include culture-based techniques, such as selective and differential media and microscopy, for identifying and enumerating specific microorganisms [34]. On the other hand, rapid methods involve applying molecular techniques, such as polymerase chain reaction (PCR), enzyme-linked immunosorbent assay (ELISA), and biosensors, which offer higher sensitivity, specificity, and reduced turnaround time compared to traditional methods [34]. Within the realm of rapid microbiological testing, one emerging technology is next-generation sequencing (NGS). NGS allows for the simultaneous identification and characterization of multiple pathogens and microorganisms in a single run, providing a comprehensive view of the food microbiome [35]. This powerful technique can help to identify potential foodborne hazards, track the source of contamination, and monitor the effectiveness of food safety interventions [36]. Moreover, NGS enables the detection of antimicrobial resistance genes, virulence factors, and other genetic markers related to food safety [37], making it a valuable tool for proactive food safety management and surveillance programs. This article will review the currently available chemical, biological, and physical food safety testing methods and discuss their advantages, disadvantages, and applications in the future.

### 2.1. Chemical Food Safety Testing Methods

Mass spectrometry, an essential analytical instrument in food chemistry, operates by ionizing chemical compounds, typically in their gaseous state, to provide detailed structural information and quantification based on their mass-to-charge ratio (m/z). The underlying theoretical framework of mass spectrometry unfolds across several distinct phases. The process commences with the ionization stage, where the analyte under investigation transforms into charged ions. Diverse ionization techniques have been devised to accommodate varying analytes, encompassing Electron Ionization (EI), Matrix-Assisted Laser Desorption Ionization (MALDI), and Electrospray Ionization (ESI), among others. After ionization, the ions are segregated and differentiated based on their mass-to-charge ratio in the mass analyzer. Commonly utilized mass analyzers comprise Quadrupole Mass Analyzers, Time-of-Flight (TOF) Analyzers, and Orbitrap Mass Analyzers. Each of these analyzers presents its unique advantages and is suitable for divergent types of analysis. The final phase entails the detection of the separated ions and the determination of their relative abundance. The detection phase hinges on the transformation of the ions back into an electrical signal by the detector, enabling further processing and analysis. Capitalizing on the unique characteristics inherent to each stage, mass spectrometry affords precise, sensitive, and comprehensive analysis. This allows researchers to identify and quantify trace amounts of chemical substances in food samples. However, it is essential to note that specific ionization techniques, analyzers, and detectors may be required for each analyte to maximize the quality of the resultant data.

High-Performance Liquid Chromatography (HPLC), Gas Chromatography (GC), Tandem Mass Spectrometry (MS), Atomic Absorption Spectroscopy (AAS), and Ultraviolet-Visible Spectroscopy (UV-Vis) are widely employed chemical–food safety testing techniques [38]. These methods offer high sensitivity, high precision, rapid analysis, broad applicability, and the capability to detect multiple harmful substances, making them essential for protecting consumers from trace harmful substances and ensuring the safety of various food products. HPLC and GC are techniques used to separate and detect chemical components in food. While HPLC is more suitable for high boiling point, low volatility, high molecular weight [39], and diverse polarity organic compounds, GC excels at analyzing low molecular weight, thermally stable, and volatile substances [40]. Tandem MS can be combined with HPLC and GC to provide qualitative and quantitative detection of trace compounds, enhancing the sensitivity and selectivity of the analysis [41]. AAS is employed for detecting metal element content in food, boasting high selectivity and low detection limits. At the same time, UV-Vis is used for detecting chemical substances and some microorganisms, with advantages such as ease of operation and low cost [42].

**Figure 2:**
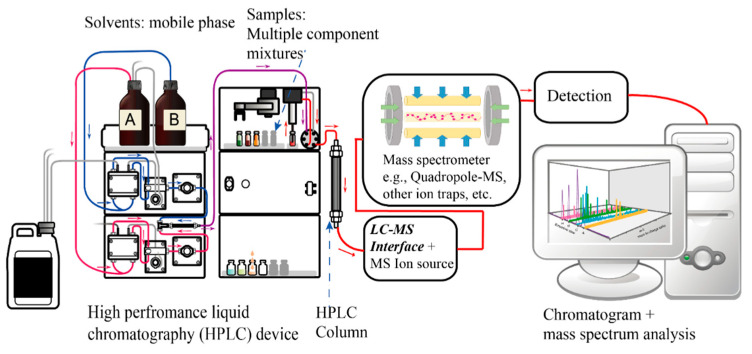
A diagram of an HPLC-Mass spectrometry system.

However, these chemical methods also present some limitations, such as the inability to detect unknown harmful substances; the operation and maintenance of mass spectrometers necessitate a significant degree of specialized knowledge and skills, as shown in Figure 2, which escalates the complexity and cost associated with their use. Furthermore, despite the high sensitivity of mass spectrometers, achieving optimal detection results may demand intricate pre-processing steps, thereby rendering the sample preparation process complex and time-consuming. In addition, mass spectrometers may yield false-positive results (e.g., residue of dithiocarbamates in organic vegetables originating from the endogenous glucosinolates in cruciferous plants) or false-negative results (e.g., epichlorohydrin converting to dichloroethanol) when detecting specific compounds, which calls their accuracy into question [43]. In the process of pesticide residue detection in food, especially when testing for fungicidal dithiocarbamates, a procedure involving the addition of hydrochloric acid is adopted, which results in the production of carbon disulfide gas. The concentration of dithiocarbamates is, then, estimated by detecting this gas. However, the acidification process generates carbon disulfide from dithiocarbamates and the naturally occurring glucosinolates in vegetables. This overlap could lead to false-positive outcomes in pesticide residue detection tests, particularly when testing for fungicidal dithiocarbamates, if the detection is solely based on carbon disulfide gas. To overcome this challenge, we require a method to directly analyze both glucosinolates within the vegetable and dithiocarbamates on the vegetable’s surface. Raman spectroscopy analysis offers a solution that directly detects dithiocarbamates on the vegetable’s surface rather than merely detecting carbon disulfide gas. Ethylene oxide in food, due to its high propensity to convert into 2-chloroethanol, cannot be directly measured by mass spectrometry. Instead, we must indirectly estimate the concentration of ethylene oxide by monitoring the level of 2-chloroethanol. However, the certainty of this conversion efficiency and the existence of other factors that may lead to the formation of 2-chloroethanol—not derived from ethylene oxide in food—are yet to be conclusively established. Despite these drawbacks, chemical–food safety testing methods play a crucial role in the comprehensive assessment of food quality and safety, making them indispensable tools for researchers and regulatory agencies worldwide.

### 2.2. Biological Food Safety Testing Methods

Foodborne diseases pose a significant threat to public health, making it imperative to ensure the safety and quality of food products. Biological food safety testing methods are essential for detecting harmful pathogens, spoilage organisms, and other undesirable microorganisms in food products [44]. This part provides an overview of conventional and rapid methods, as well as emerging technologies, with their advantages and disadvantages.

Conventional methods, including culture-based techniques and microscopy, have been widely used for a long time, and they provide a solid foundation for food safety testing. These methods are known for their broad applicability, low cost, straightforward interpretation, and reliability [45]. However, they also have some limitations, such as being time-consuming, labor-intensive, possessing limited sensitivity, susceptibility to human error, and generating significant waste [46]. Rapid methods, which employ molecular techniques such as PCR, ELISA, and biosensors, offer several advantages over conventional methods. These include higher speed, sensitivity, specificity, automation potential, and flexibility. However, despite these benefits, rapid methods may have higher costs, require technical expertise, and exhibit limited applicability. Additionally, they can be susceptible to inhibitors and have greater complexity, necessitating robust protocols and quality control measures [46]. NGS, an emerging technology in food safety microbiological testing, allows for comprehensive analysis, high-throughput processing, advanced detection, traceability, and continuous improvement [47]. NGS can simultaneously identify and characterize multiple pathogens and microorganisms, providing a detailed view of the food microbiome. However, it also has some disadvantages, including high costs, a complex data analysis, labor-intensive sample preparation, species identification limitations, and technical expertise requirement [48].

In conclusion, biological food safety testing methods are crucial in safeguarding public health by ensuring the absence of harmful microorganisms in food products. A comprehensive approach to food safety testing, combining conventional, rapid, and emerging techniques, such as NGS, enables the detection of a wide range of biological hazards. Furthermore, by understanding the advantages and disadvantages of these methods, researchers and food safety professionals can select the most appropriate tools and techniques for their specific needs [49].

### 2.3. Physical Food Safety Testing Methods

Physical food safety inspection methods are essential for detecting contaminants and ensuring the quality of food products. These methods include X-ray inspection, magnetic resonance imaging (MRI), ultrasonic testing, laser inspection, and ultraviolet (UV) inspection [50].

X-ray inspection is a powerful technique for detecting substances such as metals, plastics, and glass within packaged food items, providing high sensitivity for the detection of defects that may not be visible externally. This method is suitable for inspecting various food types, including liquids, solids, and powders, and offers fast and continuous inspection to increase productivity. High-quality images generated by X-ray inspection facilitate analysis and evaluation without causing food contamination or requiring chemical agents [51]. However, X-ray inspection is limited by its high equipment cost, poor performance with high-density foods, potential nutrient damage due to radiation exposure, demands for specialized operators, and high electricity consumption [52]. MRI is a non-destructive technique capable of detecting internal defects, such as air bubbles and cracks, in food products while having a minimal environmental impact. However, despite its high accuracy and ability to detect low-density materials such as glass and plastic, MRI is limited by its expensive equipment and maintenance costs, slow inspection speed, and potential interference with other equipment due to its magnetic field requirements [53]. Additionally, MRI is constrained by product size and shape, and it may negatively affect products with metal components [54].

Ultrasonic testing is a non-invasive, highly accurate, and rapid method for measuring the physical properties of foods such as density, hardness, and elasticity. This method offers high repeatability and non-toxic measurements. However, it is susceptible to environmental factors and requires skilled operators. Moreover, the food quality may influence the testing results [55]. UV inspection is a rapid, accurate, and simple method for detecting contaminants in food products by leveraging substances’ absorption and emission characteristics under UV light [56]. Despite its high reliability, UV inspection is prone to background interference, requires specialized equipment, cannot measure depth, and may be affected by the food’s state or composition [57]. Laser inspection is a non-destructive technique that uses high-energy laser beams to detect contaminants in food products. It is fast, efficient, and offers high-resolution imaging without needing consumables. However, laser inspection is sensitive to environmental conditions, requires expensive hardware and skilled operators, and is limited to surface defect detection. It is also inapplicable to certain food types. Furthermore, complex data processing and specialized software and equipment are necessary [58].

We will summarize and consolidate the various detection tools mentioned earlier for their applications in food safety [59,60,61]. The choice of technique for food safety inspection depends on several factors, including detection speed, detection cost, sensitivity, specificity, detection limit, and portability, as shown in Table 1 [62]. Mass spectrometry is a powerful technique that can detect a wide range of analytes with high sensitivity and specificity. However, it is also expensive, complex to operate and maintain, and requires specialized training [63]. Immunoassay is a less powerful technique than mass spectrometry, but it is more rapid and easy to use. It is also less expensive and can be used by non-specialists [64]. Spectroscopy is a versatile technique that can be used for a wide range of analytes, but it could be time-consuming and labor-intensive if there is not a high-resolution spectrometer [65]. The best technique for food safety inspection will depend on the specific needs of the application. If speed and cost are important factors, immunoassay may be a good choice. If sensitivity and specificity are important factors, mass spectrometry may be a better choice. If portability is an important factor, spectroscopy may be a better choice.

**Figure 3:**
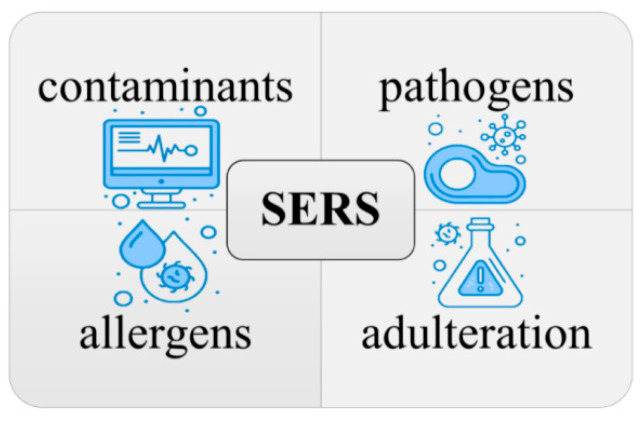
Utilizing SERS for the detection and identification of contaminants, pathogens, allergens, and adulteration in food safety.

Surface-enhanced Raman spectroscopy (SERS) is a promising technique for food safety testing. It is highly sensitive, rapid, non-destructive, and versatile, and it can be used to detect a wide range of contaminants, including food-borne pathogens, contaminants (pesticides, heavy metals, and antibiotics), adulteration, and allergens [66], as shown in Figure 3. SERS has been applied to food safety in several ways, and there is a growing body of research on its use in this field [11,67]. However, some challenges are associated with using SERS for food safety testing, such as the need to develop SERS substrates that are specific for detecting the target contaminants [68] and the need to standardize SERS protocols to ensure that results are comparable between different laboratories. The key lies in the close relationship between the microstructure of SERS and these challenges. Despite these challenges, SERS is a highly promising and complementary technique to conventional chemical analysis in the field of food safety testing.

## 3. Design, Fabrication, and Applications of SERS

**Figure 4:**
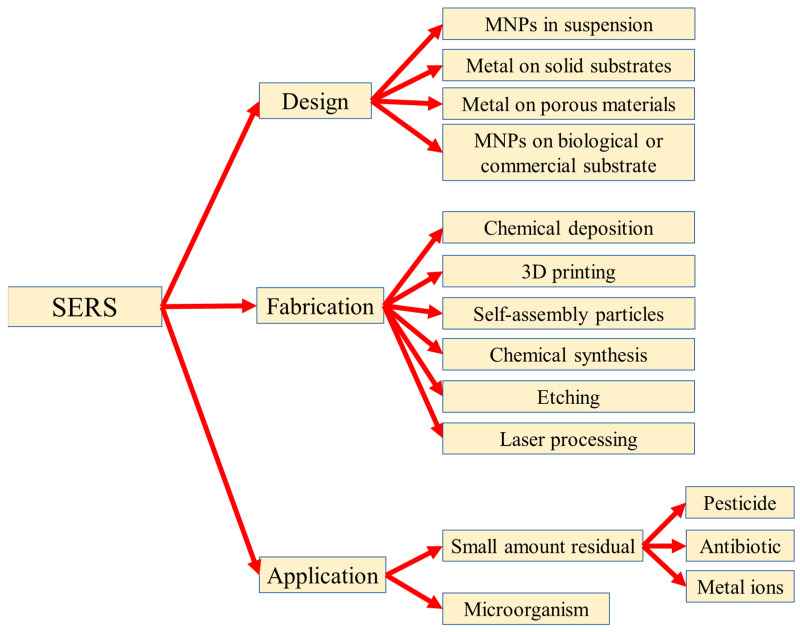
Reported design, fabrication, and the application of the SERS technique in food safety.

SERS is a powerful and sensitive method for detecting the hazardous materials in food, water, and the environment because of the advantages of the sensitive, simple, non-invasive, and low-interference water properties. In this chapter, the design and fabrication of the SERS substrates and the SERS applications for food safety are reviewed, as shown in Figure 4. In Section 3.1, the SERS substrates with four kinds of different designs were reviewed: metal nanoparticles (MNPs) in suspension, metal on solid substrates, metal on porous materials, and MNPs on biological or commercial substrates; in Section 3.2, the SERS substrates are separated into six kinds by the different fabrication processes: chemical deposition, 3D printing, self-assembly particles, chemical synthesis, etching, and laser processing; in Section 3.3, the SERS substrates are introduced by the analysis in food safety: a small amount of residue included the pesticide, antibiotic, and the metal ions, as well as the tiny biomaterials.

**Figure 5:**
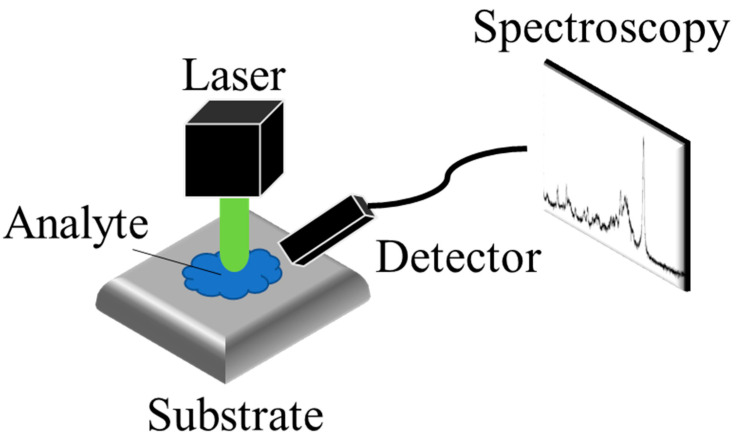
The schematic of SERS detection. The Raman scattering of the analyte, enhanced by the substrate, is detected by the detector.

**Figure 6:**
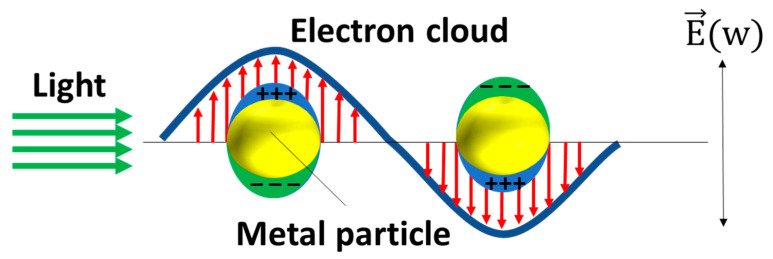
The schematic of the plasmonic resonance of the MNPs. The red arrows stand for the direction of the electron cloud and the green arrows stand for the direction of the light.

Raman spectroscopy is an analytical technique that detects the inelastic scattering of photons to observe the vibrational and rotational modes of molecules. This interaction between photons and molecules provides a unique spectroscopic “fingerprint” that can be used to identify different molecular species. As Figure 5 shows, SERS was first discovered in 1974 by Fleischmann et al. [69] when they observed a significant enhancement of the Raman signal from pyridine molecules adsorbed on a rough silver electrode [69]. The enhancement in SERS can be attributed to two mechanisms: electromagnetic mechanisms (EM) [70,71] and chemical mechanisms (CM) [72,73]. The EM arises from the increased local electric field at the metal surface, which occurs due to the generation of local surface plasmon resonance (LSPR) when the metal is illuminated with light. Surface plasmons refer to the collective oscillations of unbound electrons within a region confined to the interface between a metal and a dielectric material. As Figure 6 shows, the free electrons within the metal nanostructures oscillate in response to the incident light, and these oscillations are confined to the isolated nanostructures, leading to the resonance of the electrons and a subsequent increase in the local electric field. The enhancement factor of the surface-enhanced Raman spectroscopy (SERS) substrate can be estimated using the approximate formula *EF* ≈ (|*E*_*loc*_|/|*E*_0_|)^4^, taking into account a squared factor for the excitation and another squared factor for the emission [74]. The significant amplification of the local enhanced electric field contributes to the enhancement of the SERS signal, leading to the major development of EM techniques in SERS applications.

The CM, on the other hand, arises from the charge transfer (CT) between the analyte molecule and the substrate. This charge transfer can increase the Raman cross-section and, consequently, enhance the intensity of the Raman signal. Lombardi and Brike elucidated the Herzberg–Teller surface selection rules as a means to elucidate the role of CT, which could enhance the intensities of non-totally symmetric modes, thereby exhibiting a pronounced dependence on wavelength or voltage [75,76]. Recently, several research groups have undertaken comprehensive investigations into the surface enhancement of semiconductor materials [77,78,79]. In general, the SERS of inorganic semiconductors is primarily attributed to chemical enhancement and, specifically, the contribution of CT, resulting in enhanced molecular signals. Certain researchers have directed their efforts towards studying the SERS mechanism by examining CT states within semiconductor materials [77,78,79]. Numerous studies have demonstrated the effectiveness of investigating carrier distribution, density, and motion trends as a strategy for understanding the SERS mechanism, thereby enhancing the theoretical understanding of semiconductor-based SERS [77,78,79]. Drawing from prior discussions, the design and fabrication of SERS substrates play a crucial role in achieving the enhancement effect. Overall, SERS provides a powerful platform for highly sensitive molecular detection and analysis, and its enhancement capabilities heavily rely on the careful design and fabrication of SERS substrates.

### 3.1. The Design of the SERS Substrates

The selection of substrates in SERS technology is critical for achieving optimal sensing performance. The design of SERS substrates should be tailored to the specific target substances. In food safety applications, the target substances can be categorized into two groups: small amount molecules [80,81,82,83,84,85,86,87,88] and tiny biomaterials, such as cells [89,90,91,92], cell walls [93,94,95,96], and spores [97,98,99,100]. The key distinction between these two categories lies in their size. Molecules are typically smaller than 100 nm and can be accommodated within the hotspot of the SERS substrate [80,81,82,83,84,85,86,87,88]. On the other hand, tiny biomaterials are generally larger than 100 nm and cannot fit into the small hotspot of the SERS substrate [89,90,91,92,93,94,95,96,97,98,99,100]. Therefore, the size of the target substance is a crucial factor to consider when designing a SERS substrate for different target substances. In this review article, we will provide a detailed introduction to the design of SERS substrates for small molecules, including the mechanisms, materials, and processes involved. SERS substrates for tiny biomaterials will be briefly mentioned in Section 3.3, as they are often associated with biotechnology applications, such as the use of aptamers or antibodies, which are beyond the scope of this article. Table 2 presents various designs of SERS substrates.

The choice of SERS substrate significantly affects the Raman enhancement, prompting researchers to invest in the development of diverse substrates for SERS measurements over the past few decades. These SERS substrates can generally be classified into two categories: metal nanoparticles (MNPs) in suspension and metal nanostructures on solid substrates. The utilization of MNPs in suspension offers several advantages for SERS applications, including easier chemical synthesis (typically without the need for complex equipment) and the production of excellent enhanced Raman signals. For instance, after Kneipp first used colloidal silver nanoparticle solution to detect a single R6G molecule in 1997 [101], subsequent studies have demonstrated the achievement of single-molecule detection using nanometal colloids. As a result, ongoing efforts focus on the development of metal nanoparticles, including different shapes of MNPs [102,103,104,105,106,107,108,109,110], composite materials of MNPs [111,112,113,114,115], and the modified MNPs [116,117,118].

**Figure 7:**
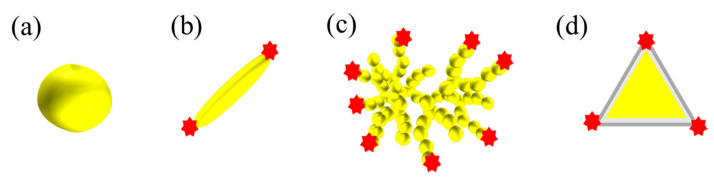
The reported MNPs with various shapes of (**a**) spheres, (**b**) rods, (**c**) branches, and (**d**) triangular nanoprisms, as well as the anisotropic hotspot.

Currently, various shapes of metal MNPs have been proposed, including the nanorods [111,116], nanostars [109], nanocubes [110], hyperbranched Au nanocorals [102], flower-like Au NPs [103], Pt-Au triangular nanoprisms [104], Au popcorns [105], decahedral Ag NPs [106], Au nanofoams [107], and dual-gap Au nanodumbbells [108]. These different shapes are aimed at creating more branches, pores, or tips on the MNPs, which are believed to generate high-intensity electromagnetic regions, also known as anisotropic hot spots. As shown in Figure 7, compared to nanospheres with isotropic geometry, nanorods, nanobranches, and triangular nanoprisms exhibit anisotropic hotspots on the tips. Additionally, composite materials have been proposed to achieve specific properties. For instance, the magnetic composite NPs, such as the Fe_3_O_4_@Au nanorod shell [111], Fe_3_O_4_/Cu_2_O-Ag nanocomposites [112], and Fe_3_O_4_/ZrO_2_/Ag composite microsphere [113], can separate the target analytes to minimize matrix effects and maximize enhancement during detection. Another composite strategy aims to improve the stability and dispersion of Ag nanoparticles. While Ag NPs have the strongest plasmonic resonance, their instability often leads to aggregation or oxidation due to high surface free energy. Therefore, composite MNPs, such as Ag NPs covered by SiO_2_ shells [114] and the Au-Ag alloy nanoparticles synthesized by the laser [115], have been proposed to enhance the stability of Ag NPs. Furthermore, modification or functionalization of MNPs can enable selective detection [117], control of nanoparticle size [117], and the generation of large Raman scattering cross-sections [118]. Examples include the use of epsilon-Poly-L-lysine-conjugated Au nanorods [116], AuNPs coated with Prussian blue [117], and boric acid-functionalized Ag NPs [118]. However, there are also disadvantages associated with colloid MNP SERS substrates. For instance, it can be challenging to control the enhancement factor of suspensions, as it is difficult to prepare suspensions containing uniformly sized nanoparticles. Moreover, SERS often requires a higher density of metal nanoparticles for practical use. However, the high surface activity of metal nanoparticles leads to their agglomeration and cluster formation before measurement, resulting in the instability of MNPs in suspension and difficulties in signal reproducibility.

**Figure 8:**
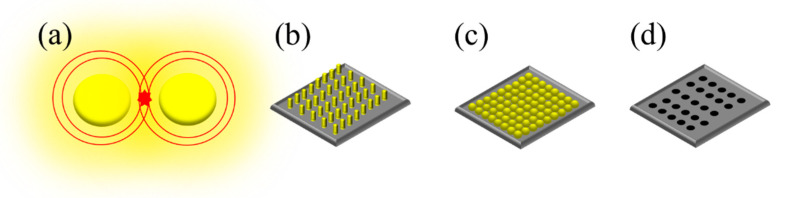
The different types of the SERS substrates: (**a**) nanoparticles, (**b**) nanopillar, (**c**) micro-nanosphere, and (**d**) nanopores.

To overcome the limitations of MNPs in suspensions, solid substrates have been employed as supporters in SERS sensing. This approach addresses the issues of uniformity, reproducibility, and stability encountered with MNPs in suspensions. Solid substrates offer well-defined nanostructures that ensure the uniform and stable distribution of MNPs. As presented in Table 2, various nanostructured solid substrates have been explored, including the Si micropyramid [119], Si nanowires [120,121], TiO_2_ nanorods [122], polystyrene (PS) beads [123], microneedle [124], ZnO nanopillar [125], CuO nanorods [126], and UV curable resin [127]. In Figure 8a, it can be observed that MNPs generate hotspots in the nanogaps between them. However, controlling the gap distance between each MNP remains a challenge. To address this, fixed nanostructures such as nanopillars, nanospheres, and nanopores are utilized as supporters to define the positions of MNPs and control the uniformity of hotspots. The hotspots of the nanopillar in Figure 8b [125] arise from the narrow gap between the nanopillars, along with the overlap of localized surface plasmonic resonance. The regularity of the nanopillar leads to a notable enhancement in the uniformity of hotspot distribution. In Figure 8c,d, the hotspots of the micro-nanosphere [123] and nanopores [81,128,129,130,131,135,136,137] are typically generated by attaching MNPs onto their nanostructures, serving as support structures to maintain the regularity of the MNPs.

Nanostructures such as nanorods, nanopillars, and nanowires, decorated with MNPs, have the capability to generate controllable hotspots by tuning the distance between each nanostructure. This configuration offers advantages in terms of sensitivity, uniformity, and stability. However, the fabrication processes for these nanostructures are often complex and time-consuming. Therefore, it is crucial to develop simple and facile methods for fabricating nanostructure supporters. Porous materials present another option as SERS supporters. These materials possess a large specific surface area, controllable porosity, and can be easily fabricated on a large scale. Various porous materials have been proposed, including porous SiO_2_ [81], porous Si [128], porous TiO_2_ [129], porous Al_2_O_3_ [130,131,135], Cu foam [136], and Ni foam [137]. These materials serve as a platform for supporting MNPs or metal nanofilms to generate high-density electric fields. The nanopores within these materials provide additional contact points for the efficient embedding of nanoparticles, leading to the generation of numerous hotspots between them. This configuration offers improved sensitivity, uniformity, and stability. It is worth noting that our previous research proposed a new mechanism, demonstrating that the electric field can be synergistically enhanced by the peripheral effect, gaps, and tips of nanopores on an anodic aluminum oxide (AAO) template covered with a Pt film [130,131]. In comparison to SERS substrates with MNPs, the nanofilm-based SERS substrate exhibits better uniformity and stability.

To further simplify the fabrication of the SERS substrate, some biological or commercial substrates were applied to be a simple SERS substrate, such as a cuttlebone-derived organic matrix [98], diatomite [138], beetle wings [139], cotton swabs [140,141], and filter paper [142]. The biological materials usually have complex structures such as the “wall-septa” on the cuttlebone [98], 3D periodic microstructures on the beetle wings, and the micro porous structure on the diatomite are naturally formed micro-to-nano structures that can be directly decorated with MNPs [98,139] or that used to be the mold to translate the nanostructure to PDMS [139]. Other simple SERS substrate supporters are commercial substrates such as cotton swabs [140,141] and filter paper [142]. Those commercial substrates are constructed with fibers, which allows the NPs adhesion and generates the hotspots on the fibers. The fiber-based SERS substrates have the advantages of the good hydrophilicity, flexibility, and the low-cost. However, the sensitivity and uniformity of fiber-based SERS substrates are a challenge. In brief, those commercial simple SERS supporters may suffer from the lower sensitivity because they lack the well-defined nanostructures. In addition, those simple SERS substrates may show lower uniformity and stability due to the individual differences between each substrate, as well as being without a controllable method to ensure the quality of the SERS substrates. It should be noted that Table 2 provides only a rough categorization of current SERS substrates, as there exists a multitude of diverse designs and fabrication techniques for such substrates. Each substrate type may encompass numerous possible variations in SERS materials, with manufacturing processes ranging from straightforward to laborious and sensitivity varying across a wide range. Consequently, discovering a sensitive, uniform, and stable, yet simple, SERS substrate remains a significant challenge.

### 3.2. The Fabrication of the SERS Substrates

In this section, the fabrication process of SERS substrates, based on the solid supporters, such as nanostructured solid substrates and porous materials, are further reviewed. As shown in Table 3, the method of fabricating the nanostructures can be separated into two strategies: bottom-up and top-down.

**Figure 9:**
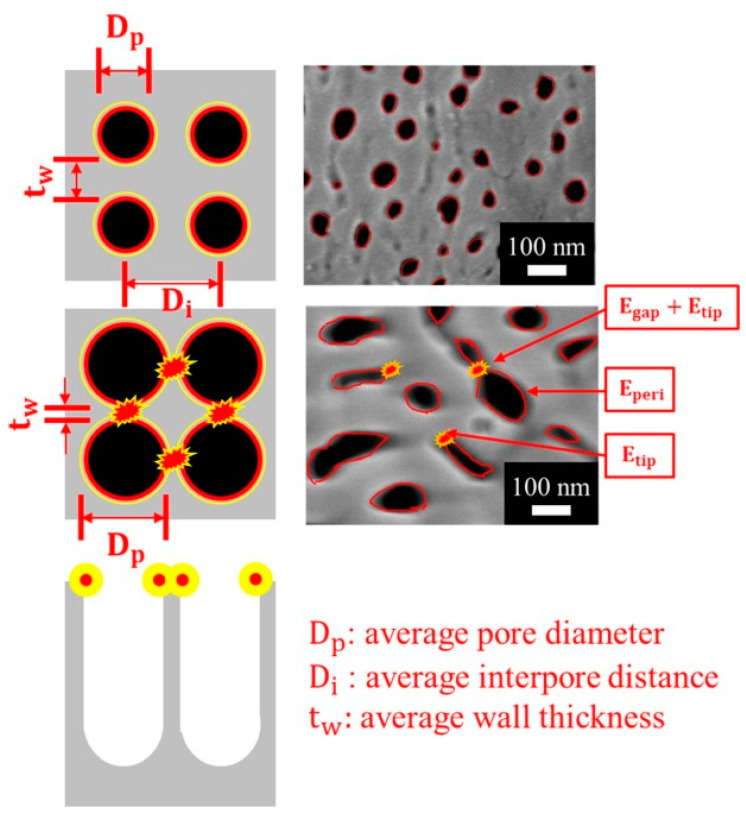
The schematic pore–peripheral–plasmonic mechanism of SERS substrate using metal-AAO-Al. The red lines around the pores are the positions where the plasmonic oscillation is the strongest, denoted as E_peri_, and the yellow lines are the boundary of the plasmonic oscillation. The small gap, t_w_ = D_p_ − D_i_, between two neighboring pores or the high-curvature tips of pores to form extra hot spots are denoted as E_gap_ and E_tip_, respectively.

In the bottom-up approaches, the nanostructures can be done by chemical deposition [120,129,143,144], 3D printing [124,145], self-assembly particles [123], and chemical synthesis [126,130,131,132,133,134,135]. During the chemical deposition, controlling the size and the shape of the deposited nanostructures is a challenge. Durastanti et al. used SiH_4_ and H_2_ as precursors to grow the Si nanowire with the length of 2–3 μm and the diameter of 40–70 nm by plasma-enhanced chemical vapor deposition (PECVD) [120]. Malik et al. deposited TiO_2_ shells on the carbon soot layer to generate the TiO_2_ fractal nanostructure [129], due to the carbon soot layer usually consisting of small particles with sizes of 1–100 nm [146]. The 3D printing has the advantage of a well-defined microstructure [147]; however, the 3D printer cannot construct the nanostructure directly, and a decent investment is necessary for high resolution 3D printers [147]. Yi et al. used 3D printing to fabricate a microneedle (MN) patch to puncture the surface of agricultural products to detect pesticide residues inside and on the surface at the same time [124]. Self-assembly particles, especially the polystyrene (PS) beads, are common materials used to form the nanostructure. The PS spheres are pushed to assemble closely by the capillary forces of the meniscus in the solvent. The flow of solvent brings in more spheres, and a large-area ordered monolayer thus grows [123]. In addition, the diameters of the PS beads are tunable, by plasma etching, to form the suitable size of SERS applications [148]. Some nanomaterials can be applied as SERS substrate supporters, such as the CuO nanorods and AAO nanopores, by chemical synthesis [125,130,131,132,133,134,135]. The two-step AAO templates with regular monodisperse pores have the tunable pore diameter and depth, so they are generally used to produce SERS substrates by depositing MNPs to generate plasmonic resonance between the MNP clusters [135]. However, the traditional two-step AAO needs a long-term fabrication process; therefore, our team developed a facile one-step anodization at room temperature, followed by pore widening, to fabricate the irregular pore with peripheral plasmonic resonance around the nanopores, as Figure 9 shows [130]. In addition, the conventional AAO fabrication was performed with cooling equipment to prevent the AAO from burning during the accumulated Joule heat, and we developed the hybrid pulse anodization (HPA) that allows the AAO to grow at room temperature without demanding the Joule heat [132,133,134].

In the top-down approaches, etching is a common method to generate nanostructures, such as Si micropyramid [119], porous Au [149], Si nanowires [121], and Si nanopillar [150]. The Si micropyramid can be simply fabricated by etching the Si wafer in the KOH due to the anisotropic properties of the Si wafer [119]. Kochylas et al. fabricated Si nanowires by metal-assisted chemical etching, and they decorated the Si nanowires with Ag nanoparticles. By tuning the etching time, the morphology of the Ag nanoparticles formed dendritic structures instead of aggregating together, which shows higher sensitivity [121]. The reactive ion etching (RIE) is a suitable option when aiming for uniform surface structure coverage over large areas. The RIE system uses a plasma source consisting of highly reactive ion species, and when they bombard the sample, a chemical reaction takes place that selectively erodes away the sample surface [150]. Kandjani et al. used RIE to fabricate the uniform and well-defined nanopillar on the silicon wafer in 18–30 min. However, the Si-based fabrication process suffers from high-pollution chemicals such as HF or SF_6_; therefore, a simple and green method on Si fabrication is still a challenge. Bai et al. used laser processing to ablate the ZnO to form the nanostrip, nanopillar array, nanogrooves, and nanocavities by using lasers with different wavelengths of 1030 and 515 nm [125]. In summary, the researchers should consider the materials, the process, and the corresponding economic benefits of the SERS substrates.

**Table 3:** The currently reported SERS substrates with different fabrication processes.

Strategy	Method	Substrates	Structure	Fabrication Time	Refs.
Bottom-up	chemical vapor deposition	Si nanowire	Si nanowire around 2–3 μm long, with an average diameter at the bottom of about 40–70 nm	-	[120,143]
		Fractal structured TiO_2_ network	TiO_2_ nano fractal structured	Form TiO_2_ for 20 min at 65 °C, and calcination process at 650 °C for 1 h in an air atmosphere.	[129]
	chemical bath deposition	MoO_3_	MoO_3_ sea urchin-like microstructures	The reaction mixture was heated to 90 °C for 3 h.	[144]
	3D printing	photosensitive resin transfer to PDMS	Micro needle: the height, bottom diameter, and top diameter of the needle are about 880, 470, and 25 μm, respectively	-	[124]
			3D arrays of pores of 2 μm in diameter and 1.4, 1.8 and 2.2 μm in depth	-	[145]
	Self-assembly particles	PS beads	1.5 μm-diameter monodisperse PS spheres	24 h for PS beads Self-assembly	[123]
	Chemical synthesis	CuO nanorods	CuO nanorods structure has a smooth surface with a diameter ranging from 50 to 100 nm, and the length of the rods is estimated to be 5 μm.	heated at 500 °C for 120 min	[126]
		AAO nanopores	46–72 nm nanopores in 4 cm^2^	3 min anodization and 10 min etching	[130]
			nanopores with 100 nm depth and a 80 nm diameter.	7 h anodization and total 5 h etching	[135]
Top-down	Chemical etching	Si micropyramid	8.4 µm Si micropyramid in 4 inches wafer	85 °C for 90 min etching in KOH	[119]
	Chemical etching	Porous Au	the width of the Au ligaments is in the range of 40 nm	copper etching of the stacked multilayers at room temperature for 300 min to form the porous Au	[149]
	Metal assisted chemical etching	Si nanowires	440–480 nm Si nanowires	Etching in the solution containing AgNO_3_,hydrofluoric acid (HF), and DI water for 6 min.	[121]
	SF_6_/O_2_ gas reactive ion etching	Si nanopillar	Si nanopillar with height of 639–2217 nm, apex thickness of 112–144 nm.	SF_6_/O_2_ gas reactive ion etching for 18–30 min.	[150]
	Laser processing	ZnO	Nanostrip, nanopillar array, nanogrooves, nanocavities.	-	[125]

### 3.3. The SERS Application on Food Safety

**Figure 10:**
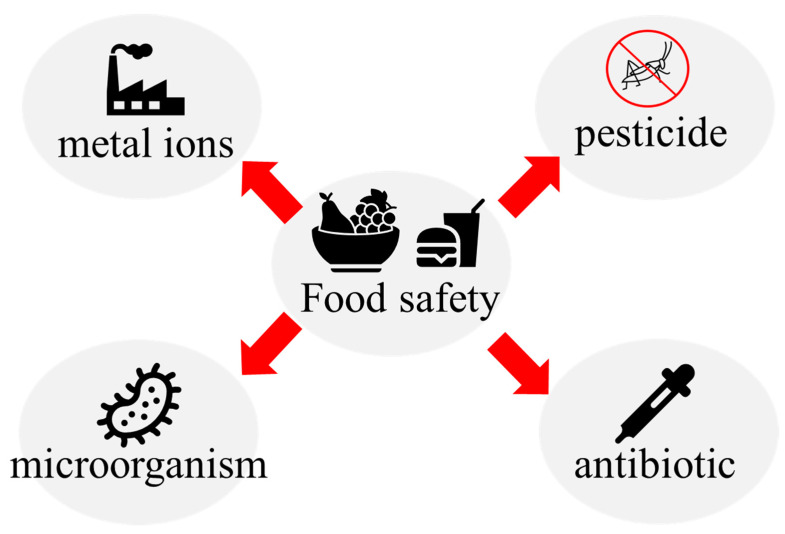
Schematic of food safety detection.

As Figure 10 shows, The SERS applications on food safety can be divided into four kinds, depending on the sensing targets: the pesticide, antibiotic, microorganic, and metal ions. To detect various analytes, SERS substrates fabricated for the different functions are required. In this section, the SERS substrates applied for food safety that included the pesticide, antibiotic, microorganic, and metal ions are reviewed, respectively.

Table 4 shows currently reported SERS substrates for sensing pesticides and their substrates, metal particles, target substances, and performances [151,152,153,154,155,156,157,158]. In the various pesticides, thiram is one of most common pesticides in SERS applications, such as detecting the thiram on the surface of apples [153,156], oranges [154], strawberries [156], mushrooms [156], tea leaves [158], and farm-produces [159]. Xiao et al. fabricated a 3D SERS substrate by reducing the gold NPs on the bacterial cellulose (BC). The BC film, coated with the gold NPs, formed a flexible nanocomposite SERS substrate to determine thiram on apple surfaces to the concentration of 0.5 ppm [153]. Liu et al. fabricated Au@Ag core-shell nanorods by the liquid–liquid interface self-assembly method and coated the Au@Ag core-shell nanorods on the silica gel to form the flexible and transparent SERS substrate [156]. The Au@Ag NRs arrays can detect the thiram on the surface of the strawberries, mushrooms, and apples with LOD of 2 μg/L [156]. Ye et al. [158] synthesized the ZnO@Co_3_O_4_ hetero-structure derived from the metal–organic framework (MOF) of ZIF-8@ZIF-67 to create the supporter with porous structures, large specific surface area, and good adsorption. The ZnO@Co_3_O_4_ hetero-structure, coated with AgNPs, can detect the triazophos, fonofos, and thiram residues in tea and dendrobium leaves with LOD of 10^−7^ M, 10^−6^ M, and 10^−6^ M, respectively [158]. Wang et al. developed an approach to construct the Ag single-atom site on the Au nanostructure array, which can modify the interfacial electronegativity near hotspots [151]. With the Ag single-atom site decoration, the acetamiprid with concentration of 0.1 ppb can be determined [151]. Those pesticides which have good affinity towards the SERS substrates can be directly detected using SERS techniques without any aptamers or linkers. However, those pesticides that have less affinity towards the SERS substrates, such as organophosphorous or organochlorine species, should be detected by selecting some aptamers, according to the characteristics of the target, to increase the signal for practical applications [152].

Antibiotics are widely applied in various fields of food safety, such as poultry production, livestock production, and aquaculture, to protect the production from microorganism infections. However, the antibiotic residues are an important issue for multiple food products, such as meat, milk, fish, and honey [160,161,162,163,164]. The antibiotic residues can enter human bodies through food chains and the bioaccumulation. High concentration antibiotic residues could damage human organs and lead the human body develop antibiotic resistances. Table 5 lists the currently reported SERS substrates for sensing antibiotics and their substrates, metal particles, target substances, and performances. Li et al. synthesized the anisotropic ZnO@Ag nanoflower by using the wet chemical synthesis method, and they assembled the ZnO@Ag nanoflower on the polyester fiber membrane to fabricate the SERS substrate for sensing florfenicol in chicken, with the concentration of 1 × 10^−5^~1 × 10^−7^ M [160]. Zhao et al. prepared a 3D CoNi-ZIFs@Ag@NF composite substrate, based on Ni foam (NF), using the electrochemical synthesis method. The 3D CoNi-ZIFs@Ag@NF composite substrate can enrich target molecules to increase the sensitivity and stabilize AgNPs to prevent AgNPs from aggregation. The tetracycline in tap water, lake water, and milk are detected from 10^−5^ to 10^−9^ M, with the recoveries ranging from 94.45% to 105.21% using the 3D CoNi-ZIFs@Ag@NF composite substrate [161]. Zhao et al. prepared the magnetic nanoassemblies, which have both magnetic separation and SERS properties, consisting of a magnetic Fe_3_O_4_ core and a bilayer plasmonic shell of AuNP and Au nanostars (AuNS). The magnetic nanoassemblies have multiple hot spots between adjacent AuNP, AuNP–AuNS, and the tips of AuNS that lead to high sensitivity for detecting the tobramycin in milk and honey at the low concentration of 50 fg/mL [163]. Yu et al. decorated the AgNPs on the two-dimensional nanomaterial Ti_3_C_2_Tx with single-stranded DNA (ssDNA) as guiding templates, which can protect the AgNPs from aggregation and improve the stability of the substrate to perform a Ti_3_C_2_Tx/NA/Ag membrane substrate. Ti_3_C_2_Tx is one of the most representative MXene materials, which has potential for SERS applications due to the advantages of the large specific surface area and the abundant surface functional groups, which can be modified to adsorb aromatic compounds. Using the Ti3C2Tx/NA/Ag membrane substrate, the trace nitrofurantoin (NFT) and ofloxacin (OFX) are simultaneously quantified at ranges of 8.0–13.7 and 42.6–49.1 μg/kg in aquatic samples, with recoveries of 88.0–107% [164].

**Figure 11:**
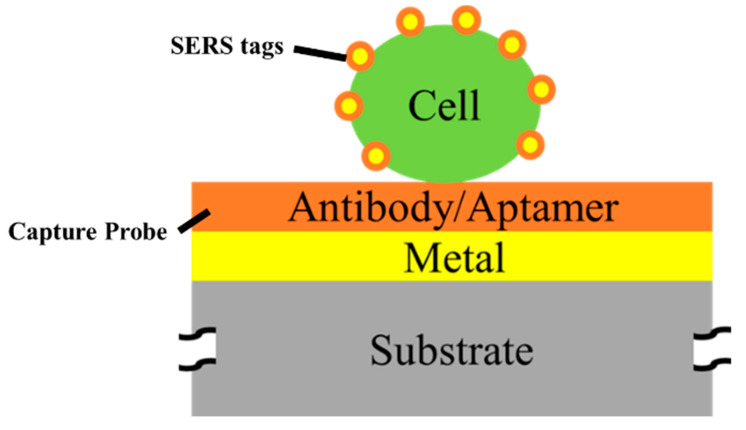
The sandwich structure for detecting the cells of microorganics (parts not to scale).

A variety of pathogenic bacteria, such as *Escherichia coli* (*E. coli*), *Staphylococcus aureus* (*S. aureus*), and *Serratia marcescens* (*S. marcescens*), can be found in raw food, and they can lead to food poisoning, tissue infections, and even death [165,166,167,168,169]. Therefore, detecting the microorganisms in food is essential. Table 6 shows the currently reported SERS substrates for sensing microorganics and their substrates, metal particles, target cells, and performances. Due to the fact that the sizes of the microorganisms are often larger than 100 nm, which cannot be fixed to a small hotspot between the MNPs, the SERS detecting microorganisms are usually designed as sandwich structures, as shown in Figure 11, which consist of capture probes, target cells, and SERS tags. The capture probes are used to fix and separate the target cells from solutions and provide aptamer binding sites to generate extra hotspots. The SERS tags can attach to the target cell and create the molecule-specific spectra to present the existence of the target cell [165,166,167,168,169]. Benesova et al. proposed a sandwich nanostructure to detect the *E. coli* using Au nanorods as the SERS tags. Au nanorods have the anisotropic hotspot in the longitudinal axis, which can strongly enhance the Raman signal of the bounding molecule on the target cell. The aptamer increases the selectivity of the SERS tags to the target cell, where the Raman signal of *E. coli* is about 3.5 times stronger than the signal of *S. marcescens* and over 28 times stronger than that of *S. aureus* [165]. Zhou et al. proposed an innovative SERS platform, based on a sandwich assay, to detect *E. coli* and *S. aureus* in milk simultaneously. Magnetic Fe_3_O_4_@SiO_2_-Au nanocrystals were used as the capture probes, which can selectively capture and enrich *E. coli* and *S. aureus*. The SiO_2_ shell prevents the Fe_3_O_4_ core from oxidation and agglomeration, and it provides abundant binding sites of aptamer, which can form numerous hot spots [166]. The graphene oxide-Au nanostars (GO-Au NSs), which have large amounts of hot spots at the tips, are used to decorate the SERS tags to generate the Raman signal for detecting the target cell. With the Fe_3_O_4_@SiO_2_-Au/cell/GO-Au NSs sandwich structure, the 10^2^ to 10^4^ cfu/mL of *E. coli* and *S. aureus* in the milk sample is detected simultaneously, with the recovery rates ranging from 92.8% to 111.1% and 91.2% to 104.9%, respectively [166]. Xu et al. coupled the hybridization chain reaction (HCR) with SERS for sensing *S. aureus*. HCR is a nucleic acid amplification strategy without an enzyme, which can realize the signal amplification. The Au-magnetic NPs, decorated with complementary DNA strands (cDNA), are used as the capture probes to separate the target cell, increase the selectivity, and realize the characteristic sensing. The Au@Ag NPs, functionalized by 4-ATP, are used as the SERS tags to generate the SERS signal of *S. aureus*. A quantitative analysis for spiked milk, with 10^2^–10^5^ cfu/mL *S. aureus*, revealed a recovery rate ranging from 91% to 102% [167]. In SERS detection for microorganisms, the aptamer is usually needed; therefore, the affinity between the SERS tags and the aptamer should be considered during the researchers’ design for a SERS substrate.

Heavy metal ions, which cannot be degraded by organisms and can accumulate through the food chain, posing a great threat to human health, are widely distributed in the environment and food [170]. Therefore, the determination of heavy metal ions is a significant issue for food safety, and tremendous efforts have been made to detect various heavy metal ions. SERS is a powerful sensing technique; however, the metal ions have no Raman signal, which means that the SERS can only detect the metal ions with chelation pre-treatment. In addition, the difficult preparation of aptamer for heavy metal ions also limits the SERS application on metal detection. It is worth noting that some metal ions detections were realized by SERS recently. Table 7 lists the currently reported SERS substrates for sensing metal ions and their substrates, metal particles, target substances, and performances [170,171,172,173,174]. Yao et al. combine the Au@Ag NPs, decorated with R6G SERS sensing the DNA functionalized lateral flow strip (LFS), with thymine–Hg^2+^ –thymine (T–Hg^2+^–T) mismatch structures. The LFS can directly and specifically recognize the Hg^2+^ ions. However, the sensitivity of LFS is not good enough for a trance analysis. Therefore, the SERS, which can further enhance the sensitivity of the Hg^2+^ ions without sacrificing the simplicity of the lateral flow strips are applied to the quantitative analysis. The platform, combined with LFS and SERS detection, has a linear range from 0.05 nm to 1 nm for sensing the Hg^2+^ ions with the R^2^ = 0.989 [170]. Li et al. synthesized the monodisperse, quasi-spherical 30 nm Ag nanocrystals from the sodium citrate and silver nitrate. Then, they coated a monolayer Ag nanocrystals film on a silicon wafer by interfacial self-assembly to enhance the repeatability of SERS detection. They use 4,4′-dipyridyl (Dpy) molecules as SERS probe molecules, which leads to decreased SERS signals when the Hg^2+^ ions are added due to the combination of Dpy-Ag being replaced by Dpy-Hg^2+^. With the Dpy molecules and the Ag nanocrystal assembly film, the LOD of Hg^2+^ was 0.35 fM, and the linear detection ranges from 5 × 10^−15^ M to 1 × 10^−10^ M [171].

Song et al. introduced a paper chromatography tandem SERS (PC-SERS) detection platform for various heavy metal ions, which is realized by a sandwich structure of 4-MBA@Au-coated PC strip/heavy metal ion/4-MBA@Au nanoparticles. The paper chromatography can separate the target ions from the complex matrix by the capillary forces, which greatly enhance the sensitivity and selectivity of the detection. The 4-MBA are decorated on the AuNPs for chelating to the metal ions to generate characteristic peaks of metal ions. On the PC-SERS platform, the 1 × 10^−2^~1 × 10^−6^ M metal ions—Cd^2+^, Cu^2+^, and Ni^2+^—included in rice are, respectively, quantitatively analyzed [173]. Currently, SERS detection of metal ions is limited to a few species, such as Hg^2+^, Cd^2+^, Cu^2+^, and Ni^2+^, as metal ions do not produce a Raman signal. Therefore, it is essential to develop chelation pre-treatments for metal ions in SERS detection.

## 4. Conclusions

In this review, we present an overview of the traditional detection methods used in food safety, including standard chemical, biological and physical methods, with a particular focus on Surface-Enhanced Raman Spectroscopy (SERS). Standard chemical methods, especially the mass spectrometers, offer high sensitivity, good precision, and the capability to detect multiple harmful substances. However, the operation and maintenance necessitate a significant degree of specialized knowledge and skills, which escalates the complexity and cost associated with their use. These requirements greatly limit the availability of those methods in high-throughput screenings. High-throughput analytical instruments, underpinned by biological and physical principles, have become pivotal in food safety and quality examinations. Instruments grounded in biological principles offer rapid, precise, and efficient evaluations. In contrast, those based on physical principles leverage optics, electromagnetism, and acoustics for accurate large-scale testing, ensuring food product quality. In addition, integrating automation with these instruments further amplifies their potential, allowing for more robust and efficient assessments. Our findings suggest that concurrently using these two high-throughput analytical tools provides a comprehensive approach to overcoming food safety and quality assessment challenges. Their continued development and synergistic utilization hold significant promise for bolstering food safety standards and facilitating future advancements in this field.

SERS has emerged as a potential approach for high-throughput screening, and a detailed review of the design and fabrication of SERS substrates is presented. Over the years, various types of SERS substrates, including suspension and solid substrates, have been developed to achieve higher sensitivity or stability. During the design and fabrication of SERS substrates, it is crucial for researchers to consider the cost compared to conventional standard chemical and physical methods. SERS substrates that require time-consuming or complex processes may be impractical for real-world applications, even if they offer ultra-sensitivity. In the section covering SERS applications in food safety, we emphasize the importance of selecting appropriate materials for SERS substrates based on the analytes being detected. The affinity between the substrates and the analytes strongly influences the testing results. Particularly, pesticides with low affinity towards SERS substrates, such as the organophosphorus or organochlorine species, may require the use of linkers to amplify the signal and enhance sensitivity. Another challenge faced by SERS is the detection of metal ions due to the lack of the Raman signal. Furthermore, the false-positive to false-negative ratio is a key metric for method efficacy. The complexity emerges from the potential false-positives, for instance, in pesticide detection assays for fungicidal dithiocarbamates, where acidification yields carbon disulfide from both the pesticides and the glucosinolates intrinsically found in vegetables. Additionally, quantifying ethylene oxide necessitates further investigation due to its conversion to 2-chloroethanol, compounded by potential interferences from other 2-chloroethanol sources. Therefore, researchers should make efforts to improve the stability of SERS substrate detection results and provide data on the false-negative/false-positive ratio of SERS substrates to assess their practicality in food safety detection. Currently reported SERS substrates exhibit semi-quantitative properties, with R^2^ values ranging from 0.91 to 0.99 and recovery rates ranging from 91% to 111%. SERS is a supplemental tool in chemical analysis for enhancing the completeness and coverage of the food safety analysis system. The high sensitivity and rapid detection capabilities of SERS technology position it as a preferred candidate for preliminary screening in the “screening first and testing later” approach to food safety management in the future.

## Figures and Tables

**Table 1:** Comparison of different techniques for food safety inspection: factors and characteristics.

Technique in Food Safety	Detection Speed	Detection Cost	Sensitivity	Specificity	Detection Limit	Portability	Refs.
Chemical methods	Slow	Expensive	High	High	Low	Poor	[59]
Biological methods	Rapid	Affordable	High	Medium	High	Good	[60]
Physical methods	Rapid	Affordable	High	Medium	Medium	Good	[61]

**Table 2:** The currently reported SERS substrates with different designs.

Substrate Type	Process	Sensitivity	Uniformity	Stability	Refs.
MNPs in Suspension	Easy Only have to synthesize the MNPs	High	Poor	Poor	[101,102,103,104,105,106,107,108,109,110,111,112,113,114,115,116,117,118]
MNPs on nanostructured solid substrates	Hard Fabricate the nanostructured substrate and deposit metal MNPs, time-consuming	Fair to high	Good	Good	[119,120,121,122,123,124,125,126,127]
Metal on porous materials	Complex Depends on the materials, the fabrication generally time-consuming	Fair to high	Good	Good	[81,128,129,130,131,132,133,134,135,136,137]
MNPs on biological or commercial substrate	Easy Only have to decorate the substrate with MNPs	Fair to acceptable	Fair	Fair	[98,138,139,140,141]

**Table 4:** The currently reported SERS substrates for sensing pesticides and their substrates, metal particles, target substances, and performances.

Ref.	Substrate	Metal Particles	Aptamer	Target Substance and Matrix	LOD	Linear Range
[151]	Au nanostructure array	single-atom construction site of in-situ synthesized Ag on Au nanostructures	No	Acetamiprid and Cycocel	Acetamiprid: 0.1 ppb	Acetamiprid: 1 × 10^−4^~1 × 10^−9^ M, R^2^ = 0.91567 Cycocel: 1 × 10^−5^~1 × 10^−9^ M, R^2^ = 0.92061
[152]	Water Solution	AuNPs reduction by Fe carbon quantum dots (CDFe)	Yes	Carbendazim and Profenofos	Carbendazim: 0.03 ppb Profenofos: 6.7 ppb	Carbendazim: 0.03–0.8 ppb, R^2^ = 0.9859 Profenofos: 6.7–53 ppb, R^2^ = 0.9687
[153]	Bacterial cellulose	Gold nanoparticles (AuNPs) were produced via the reduction in chloroauric acid with sodium citrate	No	Thiram on the surface of an apple	0.5 ppm	1–20 ppm, R^2^ = 0.99
[154]	Bacterial nanocellulose	Monodispersed silver nanoparticles prepared by a size-controllable seeded-growth method.	No	Thiram on the surface of an orange	1 × 10^−8^ M	1 × 10^−4^~1 × 10^−8^ M, R^2^ = 0.91
[155]	Amino-functionalized PDMS	Ag colloids with different concentrations were prepared by concentrating or diluting the as-synthesized Ag colloid.	No	Malathion, phoxim, and thiram in the solution	Malathion: 41.46 μg/L phoxim: 15.69 μg/L Thiram: 3.62 μg/L	Malathion: 100~5000 μg/L, R^2^ = 0.9891 phoxim: 50–5000 μg/L, R^2^ = 0.9805 Thiram: 10~1000 μg/L, R^2^ = 0.9665
[156]	Silicone	Au nanorods with a thin silver layer	No	Thiram in solution, and on the surface of the strawberries, mushrooms, and apples	Thiram in solution: 1.8 μg/L Thiram on the surface of the strawberries, mushrooms, and apples: 2 μg/L	Thiram in solution: 1 × 10^−5^~1 × 10^−9^ M, R^2^ = 0.9813
[157]	PDMS	(Ti_3_C_2_T_x_)/Ag (MXene)	No	Thiabendazole on the surface of an apple	Thiabendazole on the surface of the apple: 1 × 10^−7^ g/mL	Thiabendazole on the surface of the apple: 1 × 10^−3^~1 × 10^−7^ g/mL, R^2^ = 0.980
[158]	Silicon	AgNP-modified ZnO@Co3O4 heterostructure derived from ZIF-8@ZIF-67.	No	Thiram, triazophos, and fonofos in the solution and the tea leaves and dendrobium leaves	Solution: thiram: 10^−7^ M triazophos: 10^−8^ M fonofos: 10^−7^ M tea leaves and dendrobium leaves: thiram: 10^−6^ M triazophos: 10^−7^ M fonofos: 10^−6^ M	Tea leaves and dendrobium leaves: thiram: 1 × 10^−3^~1 × 10^−6^ M, R^2^ = 0.9835 triazophos: 1 × 10^−4^~1 × 10^−7^ M, R^2^ = 0.9942 fonofos: 1 × 10^−3^~1 × 10^−6^ M, R^2^ = 0.9570

**Table 5:** The currently reported SERS substrates for sensing antibiotics and their substrates, metal particles, target substances, and performances.

Refs.	Substrate	Metal Particles	Aptamer	Substance and Matrix	LOD	Linear Range
[160]	Polyester fiber membrane	ZnO@Ag nanoflowers	No	Florfenicol (FF) in aqueous solutions and chicken pulp extract	Florfenicol (FF) in aqueous solutions: 2.23 × 10^−10^ M	Florfenicol (FF) in aqueous solutions: 1 × 10^−3^~1 × 10^−9^ M, R^2^ = 0.9861 Florfenicol (FF) in chicken pulp extract: 1 × 10^−5^~1 × 10^−7^ M
[161]	CoNi-ZIFs@Ag@NF composite substrate based on Ni foam (NF)	Ag NPs	No	Tetracycline in DI water, tap water, lake water and milk	Tetracycline (TC) in DI water: 1 × 10^−11^ M	Tetracycline (TC) in DI water: 1 × 10^−5^~1 × 10^−10^ M, R^2^ = 0.991
[162]	Au NPs colloid	4-mercaptobenzonitrile (4-MBN)-functionalized gold nanoparticles (Au NPs)	6-Carboxyl-X-Rhodamine (ROX)-labeled aptamers	Enrofloxacin in aqueous solutions and fish/chicken meat	Enrofloxacin in aqueous solutions: 0.12 nM Enrofloxacin in fish/chicken meat: 10 nM	Enrofloxacin in aqueous solutions: 1 × 10^−9^~5 × 10^−6^ M, R^2^ = 0.98
[163]	Au NPs solution	magnetic nanoparticle (Fe_3_O_4_)-gold nanoparticle (AuNP)-gold nanostar (AuNS)	Tobramycin aptamer	Tobramycin in aqueous solutions and milk/honey	Tobramycin in aqueous solutions: 0.44 fg/mL Tobramycin in milk/honey: 50 fg/mL	In aqueous solutions: 1 × 10^0^~1 × 10^5^ fg/mL, R^2^ = 0.991 in milk/honey: 5 × 10^1^~5 × 10^3^ fg/mL,
[164]	MXene (Ti_3_C_2_Tx)	Ag NPs	No	Nitrofurantoin (NFT) and ofloxacin (OFX) in fish	NFT and OFX mixed standard solution: NFT: 1.3 μg/L OFX: 1.8 μg/L	In NFT and OFX mixed standard solution: NFT: 1.3–2.5 μg/L, R^2^ = 0.9939 OFX: 1.8–3.0 μg/L, R^2^ = 0.9964

**Table 6:** The currently reported SERS substrates for sensing microorganic and their substrates, metal particles, target substances, and performances.

Ref.	Substrate	Metal Particles	Aptamer	Target Cell and Matrix	LOD	Linear Range
[165]	Sandwich-like structure and of Au film and Au NRs	Au NRs	anti-*E. coli* rabbit polyclonal	*E. coli*, *S. aureus*, and *S. marcescens* in suspensions	-	-
[166]	Sandwich-like structure of Fe_3_O_4_@SiO_2_-Au nanocomposites and Au nanostars	Graphene oxide-Au nanostars	NH2-Apt1 (Apt of *E. coli*) and NH2-Apt2 (Apt of *S. aureus*)	*E. coli* and *S. aureus* in DI water and milk.	DI water: 10 cfu/mL Milk: 10^2^ cfu/mL	DI water: 1 × 10^1^~1 × 10^8^ CFU/mL, R^2^ = 0.994, 0.972 for *E. coli* and *S. aureus*. Milk: 1 × 10^2^~1 × 10^4^ CFU/mL
[167]	Sandwich-like structure of chitosan gel (CS gel) modified with *E. coli* Apt functionalized silver nanoparticles	gold nanostar (GNS)	The *E. coli* Aptamer	*E. coli* in DI water, milk and juice	DI water: 3.46 cfu/mL	DI water: 3.2 × 10^1^~3.2 × 10^7^ CFU/mL, R^2^ = 0.99875 milk and juice: 3.5 × 10^1^~4 × 10^3^ CFU/mL
[168]	Au-magnetic NPs decorated with complementary DNA strand	Au@Ag NPs	complementary DNA strand (cDNA) of *S. aureus* Apt	*S. aureus* in DI water and milk	DI water: 0.25 cfu/mL	DI water: 2.8~2.8 × 10^6^ CFU/mL, R^2^ = 0.9829 milk: 1 × 10^2^~1 × 10^5^ CFU/mL
[169]	Au NPs	Au NPs	ROX-DNA	*E. coli* O157:H7 in tap water, drinking water, and milk	DI water: 0.3 CFU/mL tap water, drinking water, and milk: 1 × 10^3^ CFU/mL	DI water: 1 × 10^2^~1 × 10^7^ CFU/mL, R^2^ = 0.99 tap water, drinking water, and milk: 1 × 10^3^~1 × 10^5^ CFU/mL

**Table 7:** The currently reported SERS substrates for sensing metal ions and their substrates, metal particles, target substances, and performances.

Ref.	Substrate	Metal Particles	Aptamer	Substance and Matrix	LOD	Linear Range
[170]	lateral flow strip	Au@Ag NPs decorated with R6G	DNA oligonucleotide	Hg^2+^ in DI water and tea	DI water: 0.36 pM Tea: 0.35 nM	DI water: 0.05 nM~1.0 nM, R^2^ = 0.989
[171]	Si wafer coated with monolayer Ag nanocrystals	Ag nanocrystals	4,4′-dipyridyl	Hg^2+^ in Ultra-pure water and tap water	Ultra-pure water: 0.35 fM	Ultra-pure water: 5 × 10^−15^ M~1 × 10^−10^ M
[172]	Etched Si wafer	Au nanorod arrays	No	Hg^2+^ in DI, ground and lake water	DI water: 0.1 nM Ground and lake water: 10 nM	DI water: 1 × 10^−5^ M~1 × 10^−10^ M, R^2^ = 0.984~0.998
[173]	Filter paper	Au nanoisland	No	Cd^2+^, Cu^2+^, and Ni^2+^ in rice	Cd^2+^, Cu^2+^, and Ni^2+^ in rice: 1 × 10^−6^ M	Cd^2+^, Cu^2+^, and Ni^2+^ in rice: 1 × 10^−2^ ~1 × 10^−6^ M
[174]	Au@Ag NR solution	Au@Ag nanorod	No	Hg^2+^ in DI water and egg	0.001 ng/mL	In DI water: 0.005~1 ng/mL, R^2^ = 0.9974

## Data Availability

Data are the coauthors’ research results and schematic drawing.
